# Treatment of alcohol dependence with low-dose topiramate: an open-label controlled study

**DOI:** 10.1186/1471-244X-11-41

**Published:** 2011-03-14

**Authors:** Thomas Paparrigopoulos, Elias Tzavellas, Dimitris Karaiskos, Georgia Kourlaba, Ioannis Liappas

**Affiliations:** 1 Athens University Medical School, 1st Department of Psychiatry, Eginition Hospital, Athens, Greece; 2 Harokopio University of Athens, Department of Nutrition and Dietetics, Athens, Greece

## Abstract

**Background:**

GABAergic anticonvulsants have been recommended for the treatment of alcohol dependence and the prevention of relapse. Several studies have demonstrated topiramate's efficacy in improving drinking behaviour and maintaining abstinence. The objective of the present open-label controlled study was to assess efficacy and tolerability of low-dose topiramate as adjunctive treatment in alcohol dependence during the immediate post-detoxification period and during a 16-week follow-up period after alcohol withdrawal.

**Methods:**

Following a 7-10 day inpatient alcohol detoxification protocol, 90 patients were assigned to receive either topiramate (up to 75 mg per day) in addition to psychotherapeutic treatment (n = 30) or psychotherapy alone (n = 60). Symptoms of depression and anxiety, as well as craving, were monitored for 4-6 weeks immediately following detoxification on an inpatient basis. Thereafter, both groups were followed as outpatients at a weekly basis for another 4 months in order to monitor their course and abstinence from alcohol.

**Results:**

A marked improvement in depressive (p < 0.01), anxiety (p < 0.01), and obsessive-compulsive drinking symptoms (p < 0.01) was observed over the consecutive assessments in both study groups. However, individuals on topiramate fared better than controls (p < 0.01) during inpatient treatment. Moreover, during the 4-month follow up period, relapse rate was lower among patients who received topiramate (66.7%) compared to those who received no adjunctive treatment (85.5%), (p = 0.043). Time to relapse in the topiramate augmentation group was significantly longer compared to the control group (log rank test, p = 0.008). Thus, median duration of abstinence was 4 weeks for the non-medicated group whereas it reached 10 weeks for the topiramate group. No serious side effects of topiramate were recorded throughout the study.

**Conclusions:**

Low-dose topiramate as an adjunct to psychotherapeutic treatment is well tolerated and effective in reducing alcohol craving, as well as symptoms of depression and anxiety, present during the early phase of alcohol withdrawal. Furthermore, topiramate considerably helps to abstain from drinking during the first 16-week post-detoxification period.

## Background

Alcohol dependence is a multifactorial disorder influenced by interacting genetic, biological, psychological and environmental factors. Pharmacologically, alcohol is considered to be a potent central nervous system depressant and its action is mediated through multiple neurotransmitter systems, including the GABAergic, glutamatergic, dopaminergic serotoninergic, and opiatergic system. This complex neurobiological network, which is involved in the regulation of alcohol preference, intake, and the rewarding and craving components of alcohol dependence, has been the target of various pharmacological agents, albeit with only limited success. In this context, there has been a growing interest in the use of anticonvulsant medications in the field because these agents may act on the neurobiological substrate of addiction [1,2]. In this vein, the anticonvulsant topiramate has been suggested to be promising for treating alcohol dependence [3,4]. Although the mechanism is unclear, modulation of the dopamine reward pathways of the brain through antagonizing excitatory glutamate receptors at a-amino-3-hydroxy-5-methylisoxazole-4-propionic acid and kainate receptors and inhibiting dopamine release [5,6] within the mesocorticolimbic system while enhancing inhibitory GABA (by binding to a site of the GABA-A receptor) [7], has been proposed as being responsible for its effectiveness [8]. This dual action of topiramate is supposed first to lead to a dopamine decrease in the nucleus accumbens in response to alcohol ingestion, and consequently to a reduction of its rewarding/reinforcing potential, and second to minimize withdrawal symptoms by moderating the effect of chronic alcohol consumption on neural system excitability. Thus, targeting at both facets of addiction, i.e., craving and feelings of inner tension and discomfort, relapse may be less likely. However, several issues regarding dosing, duration and tolerability of treatment with topiramate have not been adequately addressed as yet.

Treatment of alcohol dependence is a two-phase process, which aims at alcohol withdrawal and subsequent long-term abstinence and relapse prevention [9]. Depending on the phase, different priorities may be set. Thus, pharmacotherapy during and immediately after detoxification may protect from withdrawal dysphoric symptoms and can reduce anxiety and symptoms of depression [10-12]. Thereafter, medication can be used to reduce craving and reward from alcohol use. GABAergic and glutamatergic medications might be promising candidates for helping during both phases of alcohol dependence treatment [13].

The objective of the present open-label controlled study was to assess the efficacy and tolerability profile of low-dose topiramate as adjunctive treatment in alcohol dependence during the immediate post-detoxification period and during a 16-week follow-up period after alcohol withdrawal. The choice of low-dose topiramate was made based on the existing, albeit limited, literature, which suggests that even low doses of this medication can be beneficial in preventing alcohol relapse [14,15,4]; moreover, a less aggressive approach with milder side-effects could be advantageous in terms of treatment adherence. A control non-medicated group of alcohol-dependent individuals was used for comparisons in terms of anxiety and depressive symptoms, craving and drinking outcome.

## Methods

### Study design - participants

The study was an open-label controlled clinical trial. Participants were assigned either to a standard alcohol detoxification group (see below) or to a topiramate augmentation group. Assignment to the topiramate augmentation group was made on a 2:1 ratio; thus, every third intake was assigned to the topiramate group. In total, 90 alcohol-dependent individuals who consecutively contacted the Drug and Alcohol Addiction Clinic of the Athens University Psychiatric Clinic at the Eginition Hospital in Athens, Greece, were enrolled in the study. Patients had to fulfil the DSM-IV-TR [16] diagnostic criteria for alcohol abuse/dependence and were admitted for inpatient alcohol detoxification. Informed consent was obtained from the participants after providing detailed information on the objectives of the study and the research/therapeutic protocol. All procedures were approved by the ethical committee of our institution. ("Committee on Medical Ethics of the Eginition Hospital" & Reference No: 1178).

The inclusion criteria were: a) age 18-65 years, b) absence of a serious physical illness (as assessed through physical examination and routine laboratory screening), c) absence of another pre- or co-existing major psychiatric disorder on the DSM-IV-TR axis I, d) absence of another drug abuse, and e) participants with affective or anxiety symptoms were not excluded from the study if concurrent with an alcohol-abusing period; individuals who fulfilled a DSM-IV-TR diagnosis of depressive or anxiety disorder (assessed through the Schedules for Clinical Assessment in Neuropsychiatry [SCAN] [17] and information obtained from a close relative) were excluded from the study if relevant criteria were met prior to the onset of alcoholism or during periods of abstinence.

Participants were assigned to two study groups: the control group (n = 60), which included subjects treated with a standard alcohol detoxification protocol, and the topiramate augmentation group (n = 30), which included patients who were additionally given topiramate (up to 75 mg/day in 2 divided doses). Topiramate was initiated at a daily dose of 25 mg, before stopping the last dose of 5 mg diazepam, and was gradually increased up to 75 mg/day over three weeks (mean dose: 55.0 ± 19.03 mg/day).

The standard alcohol detoxification protocol was initiated and completed one week (7-10 days) after admission to the ward. This protocol includes vitamin replacement (vitamins C, E and B complex) and oral administration of diazepam (30-60 mg in divided doses), with gradual taper off over a week. Thereafter, both groups were given a standard treatment program with cognitive-behavioural short-term psychotherapy of 4-6 week duration (i.e. during their inpatient treatment). After discharge, patients were assessed at a weekly basis for 4 more months in order to monitor their course and abstinence from alcohol. Assessment of abstinence from alcohol was based on self reports, but it was further cross-checked with a family member to ascertain accuracy of information. Also, serum γ-glutamyl transpeptidase (γ-GT) and an alcohol breath test at each visit were used to control abstinence. No discrepancies were observed between these measures of abstinence throughout the study. Although there is ongoing debate regarding the reliability of self-reports of alcohol consumption, it has been shown that data thus collected are a valid source of information in the case of dependent individuals [18]. Furthermore, γ-GT is considered to be a reliable marker of alcohol relapse detection [19].

From the total sample (n = 90), eighty-five subjects (n = 85) were included in the final statistical analysis because five participants from the control group had occasionally used benzodiazepines during the follow-up period on their own initiative, and this was considered as protocol violation.

### Measures

Participants were diagnosed by the Schedules for Clinical Assessment in Neuropsychiatry [SCAN] and assessed through the Composite International Diagnostic Interview [20] (CIDI; section on alcohol consumption) for their pattern of alcohol abuse, potential major life problems related to alcohol consumption and the occurrence of withdrawal symptoms in the past. All data pertaining to alcohol use were self-reported but in order to ascertain accuracy of information a relative was also interviewed to corroborate current status and psychiatric history. Furthermore, sociodemographic data (age, socioeconomic status, marital status, level of education) and previous psychiatric history (pre-existent diagnosis, medication, number of hospitalizations) were recorded. Symptoms of depression and anxiety were assessed with the Hamilton Depression Rating Scale (HDRS) [21] and the Hamilton Anxiety Rating Scale (HARS) [22]. Obsessive thoughts about alcohol use and compulsive behaviours toward drinking (facets of craving) were estimated with the Obsessive Compulsive Drinking Scale (OCDS) [23]. Overall functioning was assessed using the Global Assessment Scale (GAS) [24]. The severity of withdrawal symptoms was evaluated twice daily during the first week of alcohol withdrawal with a modified version of the Addiction Research Foundation Clinical Institute Withdrawal Assessment for Alcohol (CIWA-Ar) [25]. Adverse effects of treatment in both groups were monitored through an adapted version of the Systematic Assessment for Treatment Emergent Events (COMBINE SAFTEE) scale, which is a structured instrument for collecting adverse events adapted for clinical studies in the alcoholism field [26]. Assessments were done at three time points; initially within 48 h upon entering the program (time point 0) and subsequently at 21 ± 2 day intervals (time point 1 & 2) over the 4-6 week study period.

### Statistical analysis

The number of subjects that entered into the final analysis was eighty-five (n = 85). Independent samples t-tests were used to evaluate differences between groups in terms of symptoms of depression, anxiety, global functioning and obsessive-compulsive drinking scores at the different time points. Within groups differences were estimated with repeated measures analysis of variance (RMANOVA). For all reported values the means (± SD) were calculated. Chi-square statistics were used to compare categorical variables, as appropriate. Cox proportional hazards model was used to estimate the hazard ratio (HR) of achieving 16 weeks of continuous abstinence and the Kaplan-Meier method was applied to calculate the cumulative probability function of reaching 16 weeks of abstinence for the topiramate and the control group. The log-rank test was used to compare the cumulative probability functions. The proportional hazard assumption of Cox model was assessed through the appropriate graph. All tests were two-tailed with statistical significance set at p < 0.05. Data analysis was performed using the SPSS statistical software package (SPSS Inc. Chicago, IL, USA).

## Results

No significant differences were observed between the control and topiramate group in terms of their sociodemographic characteristics, as well as the variables related to alcohol abuse history and withdrawal symptoms during the first week of abstinence (Table 1). As regards psychopathological symptoms both groups had similarly high scores on the HDRS, HARS and OCDS, and low GAS scores upon admission (time 0), which represent a serious psychosocial impairment. A marked improvement on all these measures was observed in the two subsequent assessments (time 1 & time 2) in both study groups [depression (p < 0.01), anxiety (p < 0.01), and obsessive-compulsive drinking symptoms (p < 0.01)]. However, subjects on topiramate did significantly better than controls concerning mood improvement, i.e., anxiety and depression (p < 0.05), and craving as well (p < 0.01) (Table 2).

**Table 1:** Sociodemographic characteristics and variables related to alcohol consumption of the sample (N = 85)

	Topiramate group (N = 30)	Control group* (N = 55)
Mean age, years (± SD)	43.8 ± 8.1	46.3 ± 11.0

Sex (M, F)	M: 27, F: 3	M: 48, F: 7

Family status (S, M, D)	S: 8, M: 16, D: 6	S: 10, M: 33, D: 12

Socioeconomic status (H, M, L)	H: 1, M: 25, L: 4	H: 2, M: 39, L: 14

Educational years	7.6 ± 3.1	8.3 ± 3.8

Age at onset, years (± SD)	24.1 ± 6.2	27.3 ± 9.6

Mean alcohol consumption (gr/day)	272 ± 115	284 ± 140

Mean (CIWA-Ar) during the first week	26.1 ± 6.7	25.7 ± 6.3

M, Male; F, Female; S, Single; M, Married; D, Divorced/separated/widowed; H, High; M, Middle; L, Low. CIWA-Ar, Clinical Institute Withdrawal Assessment for Alcohol, Revised

*All comparisons between groups were non-significant.

**Table 2:** Mean scores ± SD of the various measures of psychopathology and craving at the different time points of assessment (time 0 → time 2) in the control and the topiramate augmentation group

Variable (Mean ± SD)	Group	1^st ^Assessment (time 0)	2^nd ^Assessment (time 1)	3^rd ^Assessment (time 2)
**HDRS**				

	Controls	38.7 ± 7.6	15.9 ± 8.6+	8.1 ± 6.6∞

	Topiramate	39.6 ± 5.5	12.5 ± 3.1*+	4.9 ± 3.1*∞

**HARS**				

	Controls	30.5 ± 10.2	13.9 ± 6.7+	7.1 ± 6.2∞

	Topiramate	31.8 ± 5.3	10.9 ± 3.6*+	4.3 ± 3.8*∞

**GAS**				

	Controls	46.7 ± 5.1	75.6 ± 9.3+	85.4 ± 8.5∞

	Topiramate	46.6 ± 4.7	74.3 ± 6.7+	84.3 ± 5.6∞

**OCDS**				

	Controls	37.2 ± 8.3	17.3 ± 3.9+	13.3 ± 2.8∞

	Topiramate	37.6 ± 7.8	15.3 ± 3.9*+	10.3 ± 3.1**∞

Statistics: Between groups (independent samples t-test); within groups (RMANOVA)

*Significant difference between controls and topiramate augmentation group (< 0.05)

** Significant difference between controls and topiramate augmentation group (< 0.01)

+ Significant difference between 2^nd ^and 1^st ^assessment (< 0.01)

∞ Significant difference between 3rd and 2nd assessment (< 0.01)

**Figure 1:**
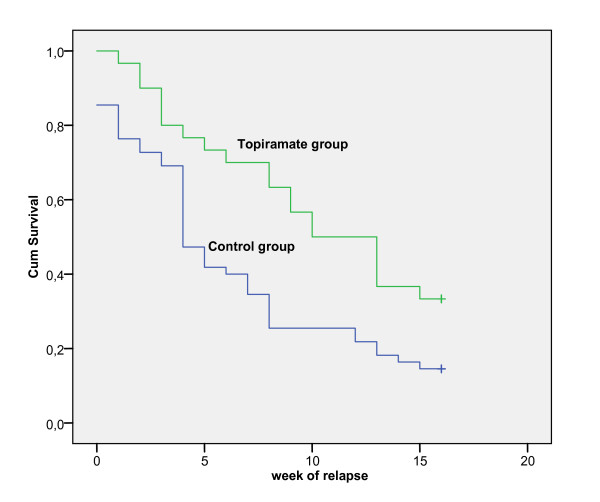
**The cumulative probability function of reaching 16 weeks of abstinence by group**.

Long-term outcome in terms of abstinence from alcohol was better for the topiramate augmentation group. Thus, although 67 patients in total (78.8%) had relapsed to alcohol use by the end of the study (16 weeks after discharge), relapse rate was significantly lower in the topiramate group (66.7%) compared with the control group (85.5%) (p = 0.043). Also, median duration of abstinence in the topiramate group was significantly longer compared to the non-medicated group (10 weeks vs. 4 weeks; log rank test, p = 0.008, Figure 1). Cox proportional hazard model showed that risk of relapse was 56% lower among patients receiving topiramate compared to controls (HR = 0.515, 95% CI: 0.304 - 0.874, p = 0.014).

Reported adverse effects are presented on Table 3. A considerable proportion (> 10%) of the topiramate augmentation group had some adverse effects, but no significant difference was recorded compared with the control group, except for somnolence which was significantly more frequent in the topiramate group (23.3% vs. 5.4%). All adverse effects were tolerable and there were no dropouts from the study.

**Table 3:** Reported adverse effects by study group

	Topiramate group N = 30 (%)	Control group N = 55 (%)
Dizziness	6 (20.0)	4 (7.2)

Somnolence	7 (23.3)	3 (5.4)*

Nervousness	2 (6.6)	7 (12.7)

Numbness/Paresthesias	3 (10.0)	3 (5.4)

Psychomotor slowness	4 (13.3)	2 (3.6)

Nausea	5 (16.6)	2 (3.6)

* Fisher's Exact Test (2-tailed sig.), p < 0.05

## Discussion

The main finding of the present study is that low-dose topiramate given as a treatment adjunct is well-accepted and effective in reducing craving for alcohol and symptoms of anxiety and depression during the early phase of alcohol withdrawal. Furthermore, topiramate combined with a psychotherapeutic intervention improves abstinence from drinking during the first 16-week post-detoxification period, in comparison with alcohol-dependent individuals receiving psychotherapy alone.

Although topiramate is not currently approved for the treatment of alcohol dependence [27], several randomized double-blind placebo-controlled trials have demonstrated its efficacy in improving drinking behaviour and maintaining abstinence[28-30]. Compared to the standard medications approved for alcohol dependence, topiramate has been found to be inferior to disulfiram in terms of days to relapse [31] and superior to naltrexone in reducing craving [32] and improving some other critical measures of drinking behaviour [30]. The precise mechanism of topiramate's favourable action is unclear. It may be that it modulates GABAergic transmission in the central amygdala, a brain region implicated in the regulation of emotionality and alcohol intake [33,34]. Also, it has been shown that GABA receptors undergo allosteric modulation by ethanol and mediate the acute and chronic effects of alcohol, including tolerance, dependence and withdrawal [35]. On the other hand, topiramate enhances the inhibitory function of GABA, antagonizes excitatory glutamate receptors, and inhibits dopamine release [36].

Topiramate has been used for the treatment of alcohol dependence in outpatient settings. Doses ranging from 150 to 300 mg/day have shown promising results, in terms of significant improvement in several dependence-related parameters [3]. However, a major concern has been topiramate's adverse effects, which are prominent especially during the titration period, appear to be dose-related but usually subside with continued treatment [37,38]. Thus, the majority of patients who discontinue topiramate treatment, due to its side effects, do so early in treatment. In this line of thought, a key objective of the present study was to establish the efficacy and side effect profile of low-dose topiramate (up to 75 mg/day) that might improve adherence to treatment. In our sample, a considerable proportion (> 10%) of the topiramate augmentation group had some adverse effects, but no significant difference was recorded compared with the control group. However, these adverse effects were tolerable and there were no dropouts from the study. This was probably due to several reasons such as that the initial detoxification took place in an inpatient basis assuring a high compliance with all treatment interventions, that the study population was highly motivated to withdraw from alcohol, and finally that our sample consisted of individuals with relatively high initial withdrawal symptoms who are usually excluded from most studies of outpatient populations.

Impulsive and compulsive behaviours play a crucial role in alcohol abuse, craving and relapse [39-41]; therefore, medications with anticraving properties have been used for prevention of relapse. Several studies have demonstrated topiramate's efficacy in the management of impulsive, aggressive and self-harmful behaviour [42], gambling [43], eating disorders [44], as well as an adjunct to SSRIs in obsessive-compulsive disorder [45]. Thus, moderation of impulsivity with consequent minimization of craving might be responsible for the lower rates of relapse in the topiramate augmentation group. Moreover, research has consistently documented a strong association between anxiety and/or symptoms of depression and alcohol abuse; these symptoms usually subside following a few weeks of abstinence [46]. However, mild anxiety [11] and minor symptoms of depression may persist for several months and various medicines - including tiagabine [47], mirtazapine and venlafaxine [12] - have been used adjunctively to standard alcohol detoxification treatment in order to increase patient compliance and improve treatment outcome [10,48]. Through such a collateral beneficial action, topiramate could lead to the more favourable outcome observed in the augmentation group of the present study.

The main limitations of the present study are: a) the relatively small sample size, which reduces the statistical significance of our findings, b) the study did not follow a double-blind placebo control design; such a design was not feasible due to the ethical restrictions of our institution, c) assessment of alcohol use during the follow-up period was mostly based on self-reports and periodically cross-checked with an informant and γ-GT measurements, and d) a longer follow-up period would provide important information on the long term efficacy of topiramate in a community setting. Despite the above limitations, our results corroborate previous reports that show the potential usefulness of topiramate in the treatment of alcohol dependence even when administered at low doses.

## Conclusions

In conclusion, low-dose topiramate when used as an adjunct to psychotherapy is well tolerated and effective in reducing alcohol craving, as well as symptoms of depression and anxiety, present during the early phase of alcohol withdrawal. Furthermore, topiramate considerably helps to abstain from drinking during the first 16-week post-detoxification period, a period which is critical for relapse. Thus, topiramate could be an alternative option beyond the already approved agents for the treatment of alcohol dependence.

## Abbreviations

GABA: γ-Aminobutyric acid; DSM-IV-TR: Diagnostic and Statistical Manual of Mental Disorders, Fourth Edition, Text Revision; SCAN: Schedules for Clinical Assessment in Neuropsychiatry; γ-GT: γ-glutamyl transpeptidase; CIDI: Composite International Diagnostic Interview; HDRS: Hamilton Depression Rating Scale; HARS: Hamilton Anxiety Rating Scale; OCDS: Obsessive Compulsive Drinking Scale; GAS: Global Assessment Scale; CIWA-Ar: Clinical Institute Withdrawal Assessment for Alcohol, revised; SAFTEE: Systematic Assessment for Treatment Emergent Events; RMANOVA: repeated measures analysis of variance; SD: standard deviation; HR: hazard ratio; SSRI: Selective serotonin reuptake inhibitor

## Competing interests

The authors declare that they have no competing interests.

## Authors' contributions

TP participated in the design of the study and drafted the manuscript; ET participated in the design of the study, data collection, and assisted in drafting the manuscript; DK participated in data collection, in the analysis and interpretation of data, and helped to draft the manuscript; IL had overall supervision of the study and made extensive revisions of the manuscript; GK participated in data analysis and their interpretation. All authors read and approved the final manuscript.

## Pre-publication history

The pre-publication history for this paper can be accessed here:

http://www.biomedcentral.com/1471-244X/11/41/prepub
